# Spectrum of CFTR gene mutations in Ecuadorian cystic fibrosis patients: the second report of the p.H609R mutation

**DOI:** 10.1002/mgg3.337

**Published:** 2017-10-11

**Authors:** Sofía C. Ortiz, Santiago J. Aguirre, Sofía Flores, Claudio Maldonado, Juan Mejía, Lilian Salinas

**Affiliations:** ^1^ Área de Biología Molecular NETLAB Laboratorios Especializados Quito Ecuador; ^2^ Departamento de Ciencias de la Vida y la Agricultura Grupo de Investigación en Sanidad Animal y Humana Universidad de las Fuerzas Armadas ESPE Sangolqui Ecuador

**Keywords:** CFTR gene, cystic fibrosis, heterogeneity, molecular diagnosis, mutation, polymorphism

## Abstract

**Background:**

High heterogeneity in the CFTR gene mutations disturbs the molecular diagnosis of cystic fibrosis (CF). In order to improve the diagnosis of CF in our country, the present study aims to define a panel of common CFTR gene mutations by sequencing 27 exons of the gene in Ecuadorian Cystic Fibrosis patients.

**Methods:**

Forty‐eight Ecuadorian individuals with suspected/confirmed CF diagnosis were included. Twenty‐seven exons of CFTR gene were sequenced to find sequence variations. Prevalence of pathogenic variations were determined and compared with other countries’ data.

**Results:**

We found 70 sequence variations. Eight of these are CF‐causing mutations: p.F508del, p.G85E, p.G330E, p.A455E, p.G970S, W1098X, R1162X, and N1303K. Also this study is the second report of p.H609R in Ecuadorian population. Mutation prevalence differences between Ecuadorian population and other Latin America countries were found.

**Conclusion:**

The panel of mutations suggested as an initial screening for the Ecuadorian population with cystic fibrosis should contain the mutations: p.F508del, p.G85E, p.G330E, p.A455E, p.G970S, W1098X, R1162X, and N1303K.

## Introduction

Cystic fibrosis (CF), an autosomal recessive disease caused by mutations in the CFTR (OMIM: 602421) gene is a multisystem impairment that involves lung damage, exocrine pancreatic insufficiency, liver disease, intestinal motility disorder, and high concentrations of sweat chlorides (Kan et al. 2015).

More than 1900 sequence variations in CFTR gene (OMIM:602421) have been reported (Derichs 2013). These variations and their prevalence vary in populations based on their geographic and ethnic origins (Hakkak et al. 2013). High heterogeneity in CFTR gene (OMIM:602421) mutations spectrum in Latin America (Orozco et al. 2000) disturbs the molecular diagnosis of cystic fibrosis in this region (Luna et al. 2007).

The incidence of cystic fibrosis in Ecuador is 1:11.252 born alive, and CFTR gene (OMIM: 602421) mutations in Ecuadorian CF patients using a kit of common mutations worldwide are: p.F508del, p.G85E, p.G542X, p.N1303K, p.G551D, and p.R334W (Valle et al. 2007). Moya et al. (2009) reported p.H609R mutation in Ecuadorian population for first time.

Ecuador has not established a common CFTR mutations panel for the diagnosis of cystic fibrosis, therefore this study aims to define this panel by sequencing the 27 exons of this gene in Ecuadorian cystic fibrosis patients.

## Materials and Methods

### Ethical compliance

This study was approved by the Hospital Eugenio Espejo Bioethics ′committee.

### Methodology

Forty‐eight Ecuadorian individuals with suspected/confirmed CF diagnosis from “Fundación Ecuatoriana de Fibrosis Quística” were included in this study. They and their legal representatives signed an informed consent for this study. The following procedures were performed at “NETLAB Laboratorios Especializados”.

In brief, genomic DNA was extracted from the individuals′ peripheral blood lymphocytes with QIAcube (Qiagen, Valencia, CA, USA). For each sample, 27 exons of CFTR gene (OMIM:602421) were amplified by PCR using the Platinum Taq DNA Polymerase (Invitrogen, Carlsbad, CA, USA) and the primers designed by Montgomery et al. (2007) in the GeneAmp PCR System 9700 (Applied Biosystems, Foster City, CA, USA).

Agarose gel electrophoresis was run to verify amplicons sizes matched with the given by Montgomery et al. (2007) for CFTR gene (OMIM:602421) exons. Then the amplicons were purified by precipitation with ethanol/sodium acetate protocol (Francis 2005).

Sequencing was performed with the Big Dye Terminator Cycle Sequencing Kit (Applied Biosystems), and the sequencing products were purified by precipitation with Etanol/EDTA protocol suggested by the Big Dye Terminator Cycle Sequencing Kit Manual.

Capillary electrophoresis was carried out in the 3500 Genetic Analyzer (Applied Biosystems). The sequences were analyzed on SeqScape 3 Software to compare them with CFTR gene (OMIM: 602421) exon sequences at NCBI database, and to find sequence variations (Table 1).

**Table 1:** Reference sequences of the 27 exons of CFTR (OMIM: 602421) gene

Exon	GenBank reference
1	M55106.1
2	M55107.1
3	M55108.1
4	M55109.1
5	M55110.1
6a	M55111.1
6b	M55111.1
7	M55112.1
8	M55113.1
9	M55114.1
10	M55115.1
11	M55116.1
12	M55117.1
13	M55118.1
14a	M55119.1
14b	M55120.1
15	M55121.1
16	M55122.1
17a	M55123.1
17b	M55124.1
18	M55125.1
19	M55126.1
20	M55127.1
21	M55128.1
22	M55129.1
23	M55130.1
24	M55131.1

## Results

### Sequence variations

We found 70 sequence variations in the 27 exons of the CFTR gene (OMIM: 602421) (Table 2) Eight of these are CF‐causing mutations: p.F508del, p.G85E, p.G330E, p.A455E, p.G970S, W1098X, R1162X, and N1303K. The p.H609R mutation was identified in 10 patients. It has been reported as probably pathogenic in one study in the Ecuadorian population by Moya et al. (2009).

**Table 2:** Sequence variations in this study for the 27 exons of the CFTR (602421) gene

Exon	Sequence variation	Type of variation (NCBI)	Amino acid change (NCBI)	Clinical significance	Number of patients
1	g.19395G>A	Unreported	Unreported	Not assigned	12
	g.19396A>C	Unreported	Unreported	Unreported	1
	g.19304delG	Unreported	Unreported	Unreported	1
	g.19304G>C	Unreported	Unreported	Benign allele^d^	1
2	g.43590T>G	Unreported	Unreported	Unreported	1
	g.43510_43511delTG	Unreported	Unreported	Unreported	1
	g.43473delG	Unreported	Unreported	Unreported	1
	g.43621T>A	Unreported	Unreported	Unreported	3
	g.43580G>T	Unreported	Unreported	Unreported	2
	g.43594A>G		Unreported	Unreported	1
	g.43583A>G	Unreported	Unreported	Unreported	1
	g.43575G>C	Unreported	Unreported	Unreported	2
	g.43582T>C	Unreported	Unreported	Untested allele^e^	1
	g.43598G>A	Unreported	Unreported	Unreported	1
	g.43592T>C	Unreported	Unreported	Untested allele^e^	10
	g.43555G>C	Unreported	Unreported	Unreported	5
	g.43544T>A	Unreported		Unreported	1
3	g.48340G>A	Missense	p.G85E	Pathogenic	5
4	g.70182A>T	Unreported	p.K114Ter	Benign^f^ (score:0.000)	2
6a	g.74655delA	Unreported	Unreported	Unreported	1
	g.74534G>C	Unreported	Unreported	Unreported	10
	g.74491G>C	Unreported	Unreported	Unreported	1
	g.74630T>C	Unreported	Unreported	Unreported	1
6b	g.75901C>T	Unreported	Unreported	Benign allele	13
	g.75923T>C	Unreported	Unreported	Unreported	6
	g.75886A>C	Unreported	Unreported	Unreported	1
	g.75896T>C	Unreported	Unreported	Unreported	1
7	g.79447C>A	Unreported	Unreported	Unreported	1
	g.79434delA	frameshift	p.G330E	Pathogenic	1
9	g.88121G>A	Unreported	Unreported	Unreported	1
	g.88012C>T	Missense	p.A455E	Pathogenic	1
10	g.98696A>G	Missense	p.M470V^a^	Polymorphism	22
	g.98808_98811delTCT	cds‐indel	p.F508del	Pathogenic	13
13	g.131210A>G	Missense	p.H609R^c^	Probably damaging^f^ (score: 0.9999)	10
	g.131478G>C	Unreported	Unreported	Unreported	1
	g.131437C>A	Unreported	Unreported	Unreported	1
	g.131851G>C	Unreported	Unreported	Unreported	2
	g.131797C>G	Unreported	Unreported	Unreported	1
	g.131653A>G	Unreported	Unreported	Unreported	1
	g.131911G>T	Unreported	Unreported	Unreported	1
14a	g.134218T>G	cds‐synom	p.T854T^b^	Polymorphism	12
	g.134337T>C	Unreported	Unreported	Unreported	1
	g.134228G>C	Unreported	Unreported	Unreported	1
14b	g.142153T>G	Unreported	Unreported	Unreported	1
	g.142152T>G	Unreported	Unreported	Unreported	2
15	g.142989G>A	cds‐synom	p.T966T^b^	Polymorphism	1
	g.143018G>T	Unreported	Unreported	Unreported	1
	g.142999 G>A	Missense	p.G970S	Pathogenic	3
	g.142934T>G	Unreported	Unreported	Unreported	1
17a	g.14998T>A	Unreported	Unreported	Unreported	3
17b	g.15095 G>A	Stop‐gain	p.W1098X	Pathogenic	2
	g.151025G>A	Unreported	Unreported	Unreported	1
	g.151010delT	Unreported	Unreported	Unreported	1
19	g.166821G>C	Unreported	Unreported	Unreported	4
	g.166824C>G	Unreported	Unreported	Unreported	1
	g.166921A>G	Unreported	Unreported	Unreported	1
	g.166880T>G	Unreported	Unreported	Unreported	4
	g.166799A>G	Missense	p.K1177R	Benign^f^ (Score:0.000)	1
	g.166754C>T	Stop‐gain	p.R1162X	Pathogenic	1
20	g.181807A>G	cds‐synom	p.P1290P^b^	Polymorphism	2
	g.181837 A>G			Unreported	1
21	g.192094C>G	Missense	p.N1303K	Pathogenic	1
22	g.204099A>C			Unreported	34
	g.203927A>T			Unreported	4
23	g.204760G>A			Unreported	2
	g.204768A>G			Unreported	2
24	g.206271 G>A	cds‐synom	p.Q1463Q^b^	Polymorphism	7
	g.206360C>A			Unreported	14
	g.206154C>T	cds‐synom	p.Y1424Y^b^	Polymorphism	1

GenBank Reference sequence for the CFTR (OMIM: 602421) gene: NG_016465.4.

a Polymorphism (Huang et al.2008).

b Polymorphism or neutral variant (Trujillano, et al., 2015).

c H609R (Moya et al. 2009).

d Benign nonpathogenic allele.

e Cases where data are not available or are unknown.

f Predicted with polyphen‐2.

Mutations with red shadow have been reported as pathogenic.

### Mutation frequencies and prevalence

The diagnosis of cystic fibrosis is established when individuals have one or more phenotypic characteristics of the disease, and evidence of abnormality in the function of the CFTR by the presence of allelic variants pathogenic CFTR (OMIM: OMIM:602421) or two values of chloride the abnormal sweat (Moskowitz et al. 2008).

According to that criteria, from the 48 individuals we took the 30 patients with pathogenic mutations, and seven patients with clinical diagnosis confirmed but with unknown clinical significance mutation (Table 4) in order to obtain mutation frequencies, so *n* = 37.

p.H609R mutation was included in pathogenic mutations because it has been reported in four Ecuadorian patients with clinical diagnosis of cystic fibrosis by Moya et al. (2009), and in our study, p.H609R was found in homozygous state in four patients with cystic fibrosis symptoms. These symptoms match with the diagnostic criteria for cystic fibrosis suggested by the World Health Organization, (2006), so p.H609R probably is a CF mutation (Table 3). Also, the software polyphen‐2 gives a score of approximately 1.00 to the p.H609R as a probably damaging mutation.

**Table 3:** Clinical data of homozygous patients for the p.H609R

Patient no	Age	Sweat test I	Sweat test II	Clinic	Diagnostic and clinical suspicion criteria according to age group. (World Health Organization)
H	9	119	No data	Chronic sinusitis and colonization by *Pseudomonas aeruginosa*	Children: Unexplained chronic respiratory symptoms *Pseudomonas aeruginosa* in bronchial secretions Chronic sinusitis Nasal polyposis Bronchiectasis
I	11	No data	No data	Lung problems	
J	25	115	131	Mild lung problems and pancreatic problems. Hospitalized for pancreatitis.	Teenagers and Adults: Chronic and suppurative unexplained lung disease and Recurrent abdominal pain Pancreatitis Distal intestinal obstruction syndrome Liver cirrhosis and portal hypertension
K	28	134	No data	Bronchiectasis. Frequently hospitalized for lung and digestive exacerbations	

**Table 4:** Variations without clinical significance in seven patients with twice positive for the sweat test

Patient	Sweat test I	Sweat test II	Mutations
A	118	98	g.19395G>A Homozygous
			c.74655del A Homozygous
B	75	112	c.43590T>G Heterozygous
			g.75901C>T Heterozygous
			c.134337T>C Homozygous
			c.166821G>C Homozygous
			g.206360C>A Homozygous
C	82	87	g.19395G>A Homozygous
			c.204099A>C Homozygous
D	76	78	c.79447C>A Heterozygous
			c.204099A>C Homozygous
E	88	90	g.19395G>A Homozygous
			c.142153T>G Heterozygous
			c.204099A>C Homozygous
			c.75923T>C Heterozygous
			c.75886A>C Heterozygous
F	80	89	p.M470V Heterozygous
			c.204099A>C Homozygous
			c.206360C>A Homozygous
G	110	89	c.206360C>A Homozygous

Allele frequencies for the pathogenic mutations were calculated (Table 5). Hypothesis test that was performed to determine the mutation frequencies in this study differ significantly from other countries (Table 6).

**Table 5:** Frequencies for the pathogenic mutations

Mutation	Absolute allele frequency	Relative allele frequency (%) prevalence
p.F508del	15	20.27
p. H609R	14	18.92
p. G85E	6	8.11
p.G970S	3	4.05
p.W1098X	2	2.70
p.G330E	2	2.70
p.A455E	1	1.35
p.R1162X	1	1.35
p.N1303K	1	1.35

There were patients with two of these mutations, so the absolute allele frequency is not the number of patients. The total allele frequency was calculated with 37 patients with two chromosomes each.

**Table 6:** Hypothesis test for the difference in proportions of pathogenic mutations frequencies (prevalences) in Ecuador and other countries

Mutation	Prevalence IC (95%)	Prevalence	Country	*P*‐value
p.F508del	20.27	37.1	Ecuador (Valle et al. 2007)	*P* < 0.05
		41.8	Colombia (Keyeux et al. 2003)	*P* < 0.05
		25	Peru (Silva 2008)	*P* > 0.05
		30.6	Chile (Lay et al. 2011)	*P* > 0.05
		40.7	Mexico (Orozco et al. 2000)	*P* < 0.05
		52.7	Spain (Bobadilla et al. 2002)	*P* < 0.05
p. H609R^a^	18.92			
p. G85E	8.11	8.9	Ecuador (Valle et al. 2007)	*P* > 0.05
		0.5	Chile (Lay et al. 2011)	*P* < 0.05
		0.5	Mexico (Orozco et al. 2000)	*P* < 0.05
		0.8	Spain (Bobadilla et al. 2002)	*P* < 0.05
p.G970S^a^	4.05			
p.W1098X	2.70			
p.R1162X	1.35	1.1	Colombia (Keyeux et al. 2003)	*P* > 0.05
		0.9	Chile (Lay et al. 2011)	*P* > 0.05
		1.6	Spain (Bobadilla et al. 2002)	*P* > 0.05
p.N1303K	1.35	2.4	Ecuador (Valle et al. 2007)	*P* > 0.05
		0.5	Colombia (Keyeux et al. 2003)	*P* > 0.05
		2.1	Mexico (Orozco et al. 2000)	*P* > 0.05
		2.5	Spain (Bobadilla et al. 2002)	*P* > 0.05
p.G330E^a^	2.70			
p.A455E^a^	1.35	8.3	Canada (De Braekeleer 1997)	*P* < 0.05

a Not found mutations in the countries: Ecuador (Valle et al. 2007), Colombia (Keyeux et al. 2003), Peru (Silva 2008), Chile (Lay et al. 2011), Mexico (Orozco et al. 2000), Spain (Bobadilla et al. 2002).

Gray color means that the frequencies in this study differ significantly from other countries (p < 0.05).

## Discussion

To the best of our knowledge, this is the first research at molecular level in Ecuador which analyzes the 27 exons of the CFTR gene (OMIM: 602421) to determine the prevalence of CFTR gene (OMIM: 602421) mutations in this population; therefore, it is an important contribution for the diagnosis of cystic fibrosis in our country.

The result of a previous study in cystic fibrosis Ecuadorian population using a CFTR common mutations kit (INNO‐LiPA CFTR 29+ Tn) was 50% of the patients without identified mutation (Valle et al. 2007). The sequencing method applied in our study allowed to find 70 sequence variations. Eight of these are CF‐causing mutations: p.F508del, p.G85E, p.G330E, p.A455E, p.G970S, W1098X, R1162X, and N1303K and probably p.H609R. The mutation detection rate of the methodology in this study is 81% in patients with confirmed diagnosis of CF. It has been demonstrated that CFTR gene sequencing has greater sensitivity relative to other molecular techniques such as hybridization, SSCP, DDGE (Dequeker et al. 2009).

The p.F508del is the most common mutation in cystic fibrosis patients worldwide, 66% (Collazo 2008). In this study, a frequency of 20.27% was detected, less than Spain and Colombia**.** The p.G85E mutation has a worldwide prevalence of 0.2% and is more common in the Mediterranean region: Spain 1%, and Italy with 1.7% (Decaestecker et al. 2004). In this study, we found a frequency of 8.11%, different from Spain, Chile, and Mexico. The prevalence of p.G85E in Ecuador is the highest in Latin America and worldwide (Valle et al. 2007) (Table 6).

The p.W1098X mutation has not been reported in other countries in Latin America or Spain, but in Turkey, had a 0.6% (Bobadilla et al. 2002) and in Israel, 1% of frequency (World Health Organization, 2006). Prevalence of the mutations p.N1303K and p. R1162X in this study are similar to other countries. The mutation p.A455E has been found in 8.3% of French–Canadian population (De Braekeleer 1997) (Table 6).

Mutation frequencies of Ecuadorian population in this study are related with the profile of CFTR gene (OMIM: 602421) mutations in a previous study (Luna et al. 2007) in Latin America, where three groups according to the frequency of pathogenic mutations are showed: (i) Argentina and Uruguay, (ii) Chile, Brazil, and Colombia, and (iii) Cuba, Ecuador, and Venezuela (Luna et al. 2007). These results confirm the difference of the CFTR gene (OMIM:602421) mutation spectrum between our country and others even inside of Latin America.

One of the most important findings of this study is the detection of the p.H609R mutation, this is the second report of this mutation in Ecuadorian population. Moya et al. (2009) found p.H609R in 4 out 6 Ecuadorian cystic fibrosis patients. These patients showed twice positive for the sweat chloride test and phenotypic characteristics of the disease.

p.H609R probably has not been included in the molecular diagnostic test for cystic fibrosis in Ecuador, because three homozygous patients for this mutation did not show any mutation in a previous panel of 89 CFTR gene (OMIM: 602421) mutations, while in one patient it was found by sequencing.

The panel of mutations suggested as an initial screening for the Ecuadorian population with cystic fibrosis should contain pathogenic mutations; eight of these are CF‐causing mutations: p.F508del, p.G85E, p.G330E, p.A455E, p.G970S, W1098X, R1162X, and N1303K found in this study.

Eleven patients were excluded for the statisticts of this study, because they had mutations that have not been reported as pathogenic. Also, they lacked of clinical confirmed cystic fibrosis. Further analysis of the clinical impact of these mutations should be determined. They have not yet been classified and the penetrance is yet unknown.

A similar study on Chilean cystic fibrosis patients concluded that sequencing of coding region and adjacent intronic segments of CFTR gene (OMIM: 602421) has a 90% detection rate, yet there are a lot of variants that have not been identified. Another factor is the certainty of the clinical diagnosis of cystic fibrosis subjected to molecular analysis. This reduces the mutation rate of this method (Lay et al. 2011).

The variations found outside the exon 1 (NG_016465.3: g.19395G> A) and exon 22 (c.204099A> C) with a rate of 15% and 48% of the study population, respectively, should continue to be evaluated to determine their effect on CFTR protein, because probably are located in intronic or regulatory regions of the CFTR gene (OMIM: 602421), as it has been described for intron 16 (Bienvenu et al. 1996) and intron 23 (Yoshimura et al. 1992), where mutations cause a change in the splicing and therefore effect on the protein.

## Conflict of Interest

We declare that there is not any conflict of interest in our research.
